# Editorial: Breast cancer resistance, biomarkers and therapeutics development in the era of artificial intelligence

**DOI:** 10.3389/fmolb.2022.1034990

**Published:** 2022-09-28

**Authors:** Abbas Khan, Liaqat Ali, Dong-Qing Wei

**Affiliations:** ^1^ Department of Bioinformatics and Biological Statistics, School of Life Sciences and Biotechnology, Shanghai Jiao Tong University, Shanghai, China; ^2^ Zhongjing Research and Industrialization Institute of Chinese Medicine, Nanyang, China; ^3^ Department of Biological Sciences, National University of Medical Sciences (NUMS), Rawalpindi, Pakistan; ^4^ Peng Cheng Laboratory, Shenzhen, China

**Keywords:** breast cancer, artificial intelligence, biomarkers, daignosis, therapeutic

Breast cancer is globally the most diagnosed form of cancer in females, and one of the major causes of death from cancer (∼2.3 million cases according to Sung et al (2021) Global cancer statistics, 2020) (Sung et al., 2021). Breast cancer is a clinical condition with distinct molecular features and genetic profiles composed of various subtypes. Triple Negative Breast Cancers are one of the most aggressive breast cancers and, particularly in comparison to other Breast Cancers, have a higher 5-years death rate after treatment. Breast cancers in young females appear to be detected at more advanced stages and exhibit more aggressive biological features compared to tumors that arise in older patients. Additionally, various factors determine the emergence of drug resistance in multifactorial diseases like breast cancer. In this regard, computational methods and the recent machine learning algorithms had exponentially increased the research against different diseases and biological processes. From virtual drug screening to the molecular mechanism and from vaccine designing to therapeutic platform development, computational approaches are of great interest. They have significantly accelerated the comprehension of genomics patterns, proteomics, structure determination, mutation stability, function correlation, and also tracing. However, our knowledge about complicated breast cancer is very limited. For instance, these methods have been previously used to understand the mechanism of drug resistance in breast cancer and discover novel biomarkers for effective treatment (Khan et al.; Khan et al., 2020b). Understanding BC mechanisms is essential to determine how the mutations help to survive by protecting themselves from the hosts’ immune defense. Computational approaches in predicting the impact of these mutations on the protein structure, function and binding to other partners offer great promise for devising therapeutic strategies. No effective and final therapeutic strategies are available. Hence, further research is needed to increase our understanding and forestall this disease. Therefore, the current study issue aims to focus on the recent advances in the development of novel biomarkers, mutation identification, and therapeutics against BC. In this special issue, 11 articles including original research articles and review articles are published with a focus on breast cancer using state-of-the-art computational methods.

In the very first study, large-scale mutational analyses have been performed to systematically screen the most damaging mutation in the recently characterized protein in breast cancer known as Pirin. Suleman et al. reported V257A, I28T, and I264S mutations as important in the context of functional variation caused by them. Using molecular simulation methods to capture the behavior of these mutations in contrast to the wild type at atomic level. They revealed a greater stability drift in the structure of PIR mediated by these mutations (Suleman et al.). Similarly, Muneer et al. used virtual drugs screening approaches to discover novel drugs against the breast cancer biomarker. Using the MPD3 database, drugs were screened against VISTA protein and validated by using molecular simulation-based approaches. From this study Three compounds, Paratocarpin K (PubChem ID: 14187087), 3-(1H-Indol-3-yl)-2-(trimethylazaniumyl)propanoate (PubChem ID: 3861164), and 2-[(5-Benzyl-4-ethyl-1,2,4-triazol-3-yl)sulfanylmethyl]-5-methyl-1,3,4-oxadiazole (PubChem ID: 6494266), having binding energies stronger than −6 kcal/mol were found to have two common hydrogen bond interactions with VISTA active site residues: Arg54 and Arg127. The dynamics of the compound–VISTA complexes were further explored to infer the binding stability of the systems. These hits can be used *in vitro* and clinical studies to determine their efficacy and usage against the BC (Muneer et al.).

In another study using the microenvironment clustering method, relapse-free survival (RFS) of different phenotypes in 100 patients with RNA sequencing-based expression data from the PATTERN trial were compared (Zhu et al.). According to this study, the microenvironment phenotypes in TNBC may be able to predict both the node-positive patients’ prognosis and the outcome of high-risk node-negative patients. Likewise, the tumor microenvironment (TME) model was proposed as the best approach for biomarker identification (Xu et al.). The other reports further demonstrated the role of single or multiple viruses in the initiation and progression of breast cancer. The review article stressed on constraining the viral-based BC spread by applying effective infectious measures (Afzal et al.). Furthermore, novel biomarkers identification and their role in the detection and progression of BC have been revealed. The review reported different biomarkers based on the type i.e. nucleic acids, proteins, or other groups. The two approaches i.e., DNA methylation and miRNA profiling were defined as the best approaches for the accurate detection of BC (Almansour et al.; Afzal et al.). In addition, the role of Frizzleds (FZDs), human receptors, has been reported in BC patients. TargetScan and miRabel target-prediction databases were used to identify the potential microRNA that regulates the expression of FZD. FZD6 was particularly identified as highly expressed among the others in BC samples. The study revealed the role of FZD6-mediated signaling of molecular silencing machinery of the Wnt pathway (Assidi et al.). Moreover, Rui et al. identified Mir-4728 as an essential biomarker for the diagnosis and prognosis of HER2-positive BC while the role of CC-chemokine receptor 7 (CCR7) and its potential druggability was explored (Alrumaihi et al.; Rui et al.). Finally, Almansour et al. explored the essential therapeutic approaches including the earlier and contemporary approaches for the treatment of BC. The review provides the choice of treatment options based on the omics technologies. The authors stressed on personalized treatment as an effective approach for better clinical outcome (Mehmood et al.). In sum, this collection piled up studies that provide a deep understanding of drug resistance, biomarkers development, diagnosis, and treatment options for the efficient management of BC. This special issue piled up essential information regarding BC drug resistance and therapeutics. This issue provides essential understanding of the key events that are responsible drug resistance, cancer progression and treament. This information can be used for structure-based and rationale based therapeutics development to control BC.
